# Evaluation of subclinical atherosclerosis in obese patients with three noninvasive methods: Arterial stiffness, carotid intima-media thickness, and biomarkers of endothelial dysfunction

**DOI:** 10.20945/2359-3997000000622

**Published:** 2023-05-10

**Authors:** Mustafa Can, Muhammet Kocabas, Zeliha Yarar, Hatice Çalışkan Burgucu, Melia Karaköse, Fatma Hümeyra Yerlikaya, Kültigin Türkmen, Mustafa Kulaksızoğlu, Feridun Karakurt

**Affiliations:** 1 Muş State Hospital Department of Endocrinology and Metabolism Muş Turkey Department of Endocrinology and Metabolism, Muş State Hospital, Muş, Turkey; 2 Tokat State Hospital Department of Endocrinology and Metabolism Tokat Turkey Department of Endocrinology and Metabolism, Tokat State Hospital, Tokat, Turkey; 3 Necmettin Erbakan University Department of Endocrinology and Metabolism Konya Turkey Department of Endocrinology and Metabolism, Meram Faculty of Medicine, Necmettin Erbakan University, Konya, Turkey; 4 Selçuk University Faculty of Medicine Department of Biochemistry Konya Turkey Department of Biochemistry, Faculty of Medicine, Selçuk University, Konya, Turkey; 5 Necmettin Erbakan University Department of Nephrology Konya Turkey Department of Nephrology, Meram Faculty of Medicine, Necmettin Erbakan University, Konya, Turkey

**Keywords:** Obesity, arterial stiffness, carotid intima-media thickness, endocan, ADAMTS7, ADAMTS9

## Abstract

**Objective:**

In this study, we aimed to evaluate subclinical atherosclerosis in patients with obesity who had cardiovascular disease risk indicators such as arterial stiffness, which is evaluated using pulse wave velocity (PWV), carotid intima-media thickness (CIMT), and biomarkers of endothelial dysfunction such as endocan, ADAMTS97, and ADAMTS9.

**Subjects and methods:**

Sixty obese subjects, including 23 subjects with body mass index (BMI) ≥ 40, 37 subjects with BMI ≥ 30 but < 40, and 60 age-and sex-matched control subjects, were included in our study. Serum endocan, ADAMTS97, and ADAMTS9 levels as well as PWV and CIMT measurements of the subjects in the obese and control groups were performed.

**Results:**

In the obesity group, PWV levels were significantly higher than they were in the control group and endocan levels were significantly lower than they were in the control group. When we compared the obese group with BMI ≥ 40 and the control group, the BMI ≥ 40 group had significantly higher PWV and CIMT levels than the control group had, whereas endocan, ADAMTS7, and ADAMTS9 levels were similar to those of the control group. When we compared the obese group with BMI ≥ 30 < 40 to the control group, endocan levels were lower in the group with BMI ≥ 30 < 40, and PWV and CIMT levels were similar to the control group.

**Conclusions:**

We found that arterial stiffness and CIMT increased in obese patients with BMI ≥ 40 and that increased arterial stiffness was associated with age, systolic blood pressure, and HBA1C. In addition, we found that the endocan levels were lower in obese patients than they were in nonobese control individuals.

## INTRODUCTION

The World Health Organization defines *obesity* as a condition in which excessive and abnormal fat accumulation poses risks to health. The definition and grading of obesity is evaluated on the basis of body mass index (BMI) and using the formula $\text{BMI}=\text{weight}(\text{kg})/\text{height}({\text{m}}^{2})$ . A BMI of ≥ 30 is considered obesity, and a BMI of ≥ 40 is considered morbid obesity. The prevalence of obesity is estimated to be 14.8% in women and 10.8% in men ( 1 ).

Obesity is associated with many comorbidities such as osteoarthritis, obstructive sleep apnoea syndrome, nonalcoholic fatty liver disease, and polycystic ovary syndrome, as well as disorders such as diabetes mellitus (DM), hypertension, dyslipidemia, and insulin resistance that increase the risk of cardiovascular disease (CVD) ( 2 ). The most important risk factors associated with increased CVD risk in obesity are endothelial dysfunction and increased central blood pressure.

Atherosclerosis is a progressive disease characterized by the accumulation of lipids, inflammatory cells, and fibrous elements in the walls of large arteries, causing progressive luminal narrowing ( 3 ). Early detection of subclinical atherosclerosis in patients without overt atherosclerosis is important to reduce the risk of CVD. A close relationship exists between arterial stiffness and atherosclerosis, and this relationship is expressed as the luminal pressure and shear stress increased by arterial stiffness, causing endothelial dysfunction, accelerating atheroma formation, and leading to the progression of atherosclerosis by stimulating excessive collagen production and deposition in the arterial wall ( 4 ). Arterial stiffness is considered one of the measurements that can detect vascular damage in the earliest period. Arterial stiffness can be assessed indirectly by measuring its velocity (pulse wave velocity [PWV]) as it travels down the artery through which the systolic pressure wave passes. Studies have shown that arterial stiffness has an independent predictive value for coronary artery disease, cardiovascular morbidity, and all-cause mortality ( 5 ). One of the techniques frequently used to evaluate subclinical atherosclerosis is the measurement of carotid intima-media thickness (CIMT). The relationship between CIMT and CVD has been demonstrated in many studies ( 6 ).

Endocan, also known as endothelial cell-specific molecule 1 (ESM-1), is an endothelial mediator with a proteoglycan structure and secreted by vascular endothelial cells. Endocan contributes to neointima formation by stimulating vascular smooth muscle cell proliferation and migration during atherogenesis. Endocan is thought to play a key role in vascular diseases, organ-specific inflammation, and endothelium-related pathological processes. Studies have shown that high serum endocan levels may be closely associated with the onset and progression of CVD ( 7 – 8 ). In some studies, endocan levels were found to be lower in overweight and obese women than they were in lean women, as well as in individuals with metabolic syndrome compared to healthy individuals ( 9 – 10 ).

A disintegrin and metalloproteinase with thrombospondin motif (ADAMTS) proteins are zinc-dependent metalloendopeptidases that are responsible for the degradation of the extracellular matrix. ADAMTS endopeptidases play important roles in many physiological and pathological processes. ADAMTS endopeptidases are involved in the degradation of extracellular matrix proteoglycans such as versican, brevican, and aggrecan, as well as in the processing of procollagen. The degradation of versican by ADAMTS endopeptidases is known to be important in the pathogenesis of atherosclerosis. Studies have shown that serum levels of ADAMTS7 and ADAMTS9 are associated with CVDs ( 11 – 12 ).

In this study, we aimed to evaluate subclinical atherosclerosis in obese patients with CVD risk indicators such as arterial stiffness (assessed by PWV measurement) and CIMT as well as serum biomarkers of endothelial dysfunction such as endocan, ADAMTS7, and ADAMTS9.

## SUBJECTS AND METHODS

This prospective study was carried out between January 2021 and December 2021 at the Necmettin Erbakan University Meram Faculty of Medicine Endocrinology and Metabolism outpatient clinic.

Sixty obese subjects, including 23 subjects with BMI ≥ 40, 37 subjects with BMI ≥ 30 but < 40, and 60 age-and sex-matched healthy control subjects with BMI < 30 were included in our study. Those younger than 18 and older than 65 years, those who were pregnant or breastfeeding, and those with known active malignancy, active infection, type 2 DM, chronic liver disease, chronic kidney disease, or atherosclerotic cardiovascular disease were excluded from the study. In addition, those with any of the obesity-related diseases such as Cushing's syndrome, hypothyroidism, and hypogonadism were also excluded from the study. Obese patients and healthy volunteers were informed in detail about the study. A written voluntary informed consent form was obtained from the patients and control subjects who agreed to participate in the study. Our study was carried out in accordance with the recommendations of the Declaration of Helsinki. The study protocol was approved by the Ethics Committee of the Necmettin Erbakan University Meram Faculty of Medicine on February 19, 2021, with approval number 2021/3099.

The height (m) and weight (kg) of patients and healthy controls were measured with underwear clothing. BMI was calculated as weight / height squared. Blood pressure was measured in both arms after a 10-minute rest and recorded as systolic blood pressure (SBP) and diastolic blood pressure (DBP).

All blood samples were taken after 8 hours of fasting, and the serum samples were separated by centrifugation and stored in deep freeze at −80 °C until analysis. Serum fasting blood glucose, fasting insulin, HBa1c, total cholesterol (total-C), high-density lipoprotein cholesterol (HDL-C), low-density lipoprotein cholesterol (LDL-C), triglyceride (TG), and c-reactive protein (CRP) levels were measured. The homeostasis model assessment insulin resistance (HOMA-IR) formula ([mg/dL×μU/mL]=fasting glucose[mg/dL]×fasting insulin[μIU/mL]/405) was used to assess insulin resistance.

We measured serum levels of endocan (catalogue number: E3160Hu, BTLab, Shanghai, China), ADAMTS7 (catalogue number: E1139Hu, BTLab, Shanghai, China), and ADAMTS9 (catalogue number: E3558Hu, BTLab, Shanghai, China) with an enzyme-linked immunosorbent assay (ELISA) reader and ELISA kits according to the manufacturer's instructions. The absorbances of the samples were measured at 450 nm using a microplate reader. Concentrations were calculated according to standard curves and expressed as pg/mL.

Oscillometric PWV measurements were performed using the brachial cuff method with a Mobil-O-Graph 24 h ABPM NG device, measuring from the upper arm region. CIMT measurements were performed using an ultrasonography device with a superficial probe from the right and left common carotid arteries and by evaluating only the posterior (distant) wall. Measurements were made by longitudinal examination from the distance between the echogenicity of the vessel lumen and the media/adventitia echogenicity. The mean CIMT was calculated as the mean of measurements from both carotid arteries.

### Statistical analysis

Statistical analysis was performed using the SPSS 22.0 program. Continuous variables are expressed as median (minimum-maximum) for nonnormally distributed data and as mean ± standard deviation for normally distributed data. When parametric test assumptions were provided, differences between independent groups were compared using the significance test of the difference between the two means (independent samples t test). In the absence of parametric test assumptions, the Mann-Whitney U test was used to compare independent group differences. In addition, Pearson and Spearman correlation analyses were used to evaluate the correlation between variables. For the differences, the P < 0.05 level was considered statistically significant.

## RESULTS

Sixty obese patients and 60 healthy control subjects were enrolled in the study. Of the 60 patients in the obese group, 36 (60%) were female and 24 (40%) were male, and their mean age was 36.12 ± 8.1 years. Of the 60 subjects in the control group, 36 (60%) were female and 24 (40%) were male, and their mean age was 34.83 ± 10.03 years. No difference was observed between the obese and control groups in terms of age and sex ( Table 1 ).

**Table 1:** Demographic and clinical characteristics of the obese group and control group

	Obese (n = 60)	Control (n = 60)	p
Age (years)	36.12 ± 8.1	34.83 ± 10.03	0.442
Gender (female/male)	36-24	36-24	1.000
BMI (kg/m²)	36.15 (30.06-53.99)	25.57 (16.14-29.8)	<0.001
Waist circumference (cm)	105 (90-141)	81 (60-103)	<0.001
Hip circumference (cm)	120.46 ± 15.16	98.56 ± 7.92	<0.001
SBP (mmHg)	126.32 ± 15.09	121.67 ± 11.51	0.060
DBP (mmHg)	83.15 ± 11.59	74.47 ± 10.51	<0.001
Glucose (mg/dL)	93.26 ± 9.07	91.71 ± 8.19	0.329
Insulin (µU/mL)	19.97 ± 14.42	12.91 ± 11.23	0.004
HOMA-IR	4.64 ± 3.57	2.94 ± 2.51	0.003
HBA1C (%)	5.46 ± 0.34	5.16 ± 0.38	<0.001
CRP (mg/L)	3.66 (0.17-22.18)	1.18 (0.20-11.25)	<0.001
Total-cholesterol (mg/dL)	179.79 ± 31.13	173.82 ± 31.57	0.299
HDL-C (mg/dL)	43.81 ± 9.84	50.88 ± 11.72	0.001
LDL-C (mg/dL)	106.17 ± 24.88	99.69 ± 30.47	0.206
TG (mg/dL)	131.30 (49.6-416)	93.70 (30-367.8)	0.001
PWV (m/s)	6.0 (4-8.5)	5.65 (4.5-8.2)	0.027
CIMT (cm)	0.056 ± 0.011	0.0551 ± 0.010	0.411
Endocan (pg/mL)	247.18(26-762)	304.57(157-577)	0.013
ADAMTS9 (pg/mL)	76.09 ± 53.62	76.18 ± 81.76	0.996
ADAMTS7 (pg/mL)	60.32 ± 92.28	93.92 ± 118.46	0.108

All values are presented as mean value ± SD or median value (minimum-maximum). ADAMTS: A disintegrin and metalloproteinase with thrombospondin motifs; BMI: body mass index; CIMT: carotid intima-media thickness; CRP: C-reactive protein; DBP: diastolic blood pressure; HbA1c: hemoglobin A1c; HOMA-IR: homeostasis model assessment insulin resistance index; SBP: systolic blood pressure; HDL-C: high density lipoprotein cholesterol; LDL-C: low density lipoprotein cholesterol; PWV: pulce wave velocity; TG: triglyceride.

BMI, DBP, HOMA-IR, HbA1c, serum insulin, CRP, and TG levels were significantly higher in the obese group compared to the control group, whereas serum HDL-C and endocan levels were significantly lower than those of the controls. Although SBP, serum glucose, total-C, and LDL-C levels were higher in the obese group than they were in the control group, serum ADAMTS7 and ADAMTS9 levels were lower than those of the control group, and no significant difference occurred between the groups in terms of these parameters. PWV was significantly higher in the obese group than it was in the control group. CIMT was higher in the obese group than it was in the control group, but we could not find a significant difference between the groups in terms of CIMT ( Table 1 ).

In the correlation analysis, a positive correlation was found between PWV and age, BMI, SBP, DBP, HbA1c, and LDL-C levels in the obese group ( Table 2 ).

**Table 2:** Results of correlation analyses for parameters thought to be associated with PWV and CIMT in obese group

	PWV			CIMT	
	r	p		r	p
Age	0.681	<0.001		-0.182	0.167
BMI	0.325	0.011		0.205	0.120
SBP	0.574	<0.001		0.257	0.047
DBP	0.471	<0.001		-0.067	0.616
HbA1c	0.283	0.030		0.210	0.113
LDL-C	0.268	0.039		0.147	0.268

BMI: body mass index; CIMT: carotid intima-media thickness; DBP: diastolic blood pressure; HbA1c: hemoglobin A1c; LDL-C: low density lipoprotein cholesterol. PWV: pulce wave velocity; SBP: systolic blood pressure. r indicates Pearson's correlation coefficient.

In the subgroup analysis of the obese group, 23 subjects had BMI ≥ 40 and 37 subjects had BMI ≥ 30 but < 40. When we compared the group with BMI ≥ 40 and the control group, SBP, DBP, HOMA-IR, HbA1c, serum insulin, CRP, and TG levels were significantly higher than they were in the control group, and serum HDL-C was significantly lower in the group with BMI ≥ 40. In the group with BMI ≥ 40, PWV and CIMT levels were significantly higher than they were in the controls ( Table 3 ).

**Table 3:** Demographic and clinical characteristics of the group with BMI ≥ 40 and the group with BMI ≥ 30 but < 40 and the control group

	BMI ≥ 40 (n = 23)	BMI ≥ 30 but <40 (n = 37)	Control (n = 60)
Age (years)	36.83 ± 7.97	35.68 ± 8.26	34.83 ± 10.03
Gender (female/male)	16/7	20/17	36-24
BMI (kg/m²)	44.1 (50.0-53.9) **	33.5(30.0-39.8) **	25.57 (16.1-29.)
Waist circumference (cm)	117 (96-141) **	97.5 (90-118) **	81 (60-103)
Hip circumference (cm)	134.78 ± 11.46 **	111.31 ± 8.79 **	98.56 ± 7.92
SBP (mmHg)	130.43 ± 11.75 **	123.76 ± 16.47	121.67 ± 11.51
DBP (mmHg)	86.65 ± 9.49 **	80.97 ± 12.35 *	75.47 ± 10.51
Glucose (mg/dL)	93.38 ± 9.63	93.18 ± 8.83	91.71 ± 8.19
Insulin (µU/mL)	24.97 ± 15.91 *	16.87 ± 12.65 *	12.91 ± 11.23
HOMA-IR	5.81 ± 4.11 **	3.91 ± 3.03	2.94 ± 2.51
HBA1C (%)	5.49 ± 0.27 **	5.45 ± 0.38 **	5.16 ± 0.38
CRP CRP (mg/L)	7.68 (1.09-22.18) **	2.70 (0.17-11.96) *	1.18 (0.20-11.25)
Total-cholesterol (mg/dL)	184.80 ± 26.95	176.67 ± 33.44	173.82 ± 31.57
HDL-C (mg/dL)	44.87 ± 9.26 *	43.14 ± 10.25 **	50.88 ± 11.72
LDL-C(mg/dL)	111.59 ± 22.90	102.80 ± 25.77	99.69 ± 30.47
TG (mg/dL)	133.6 (49,6-416)	129 (68.1-324.1) *	93.70 (30-367.8)
PWV (m/s)	6.3 (4.7-7.2) *	5.9 (4-8.5)	5.65 (4.5-8.2)
CIMT (cm)	0.0604 ± 0.010 *	0.0544 ± 0.012	0.0551 ± 0.010
Endocan (pg/mL)	289.73 (100-513)	211.01 (26-763) *	304.57 (157-577)
ADAMTS9 (pg/mL)	77.20 ± 65.73	75.42 ± 45.89	76.16 ± 81.76
ADAMTS7 (pg/mL)	57.52 ± 60.74	61.91 ± 107.03	93.92 ± 118.46

* p < 0.05

** p < 0.01

All values are presented as mean value ± SD or median value (minimum-maximum). ADAMTS: A disintegrin and metalloproteinase with thrombospondin motifs; BMI: body mass index; CIMT: carotid intima-media thickness; CRP: C-reactive protein; DBP: diastolic blood pressure; HbA1c: hemoglobin A1c; HOMA-IR: homeostasis model assessment insulin resistance index; SBP: systolic blood pressure; HDL-C: high density lipoprotein cholesterol; LDL-C: low density lipoprotein cholesterol; PWV: pulce wave velocity; TG: triglyceride.

When we compared the group with BMI ≥ 30 but < 40 and the control group, DBP, HbA1c, serum insulin and CRP levels were significantly higher, whereas serum HDL-C and endocan levels were significantly lower in the group with BMI ≥ 30 but < 40. In the group with BMI ≥ 30 but < 40, PWV and CIMT levels were similar to those of the controls ( Table 3 ).

In the correlation analysis, a positive correlation was found between PWV and age, SBP, and HbA1c levels in the group with BMI ≥ 40. No correlation was found between CIMT and any of the parameters in this group ( Table 4 ).

**Table 4:** Results of correlation analyses for parameters thought to be associated with PWV and CIMT in the group with BMI ≥ 40

	PWV			CIMT	
	r	p		r	p
Age	0.755	<0.001		0.386	0.069
BMI	0.405	0.056		0.080	0.717
SBP	0.525	0.010		-0.221	0.311
DBP	0.103	0.641		-0.012	0.955
HbA1c	0.450	0.036		0.002	0.992
LDL-C	0.350	0.101		-0.089	0.686

BMI: body mass index; CIMT: carotid intima-media thickness; DBP: diastolic blood pressure; HbA1c: hemoglobin A1c; LDL-C: low density lipoprotein cholesterol, PWV: pulce wave velocity; SBP: systolic blood pressure.

## DISCUSSION

In the current study, we found that arterial stiffness was increased in obese patients and that increased arterial stiffness was associated with age, BMI, SBP, DBP, HbA1c, and serum LDL-C levels. In the subgroup analysis performed in the obese group, we found that arterial stiffness and CIMT levels were increased in the group with BMI ≥ 40, and increased arterial stiffness was associated with age, SBP, and HbA1c levels. In addition, we found that there was no increase in PWV and CIMT levels in the group with BMI ≥ 30 but < 40. In addition, we found that endocan levels were lower in obese patients than in nonobese individuals, and ADAMTS7 and ADAMTS9 levels were not different in obese patients from nonobese individuals.

Obesity is associated with an increased risk of CVD independent of traditional cardiovascular risk factors. Each point increase in an individual's BMI above normal results in a 10% increase in the risk of atherosclerosis and coronary heart disease. Moreover, a 10 kg increase in body weight increases the risk of atherosclerotic coronary artery disease by 12% ( 13 – 15 ). Mechanisms by which obesity increases the risk of CVD include changes in body composition that can cause adverse hemodynamic effects and alter heart structure and function. Some changes in the cardiovascular system in obese patients include an increase in total blood volume as a result of increased intravascular volume due to sodium retention to meet metabolic needs, an increase in cardiac output due to increased stroke volume and heart rate due to sympathetic hyperactivation to meet metabolic demands of increased adipose tissue and lean tissue, and increased systemic vascular resistance due to low-grade inflammation, hyperinsulinism, and sympathetic hyperactivation ( 16 – 17 ). The increase in cardiac output and systemic vascular resistance also causes hypertension. All of these hemodynamic changes increase cardiac work load and lead to abnormal left ventricular geometry and predisposition to adverse cardiac remodelling ( 18 ). Leptin produced by dysfunctional obese adipose tissue (hyperplasic and hypertrophic adipocytes) in obese individuals leads to the production of proinflammatory adipokines and cytokines such as tumour necrosis factor α (TNF-α), interleukin 6 (IL-6), interleukin 18 (IL-18), resistin, retinol binding protein 4 (RBP-4), and lipocalin 2 ( 8 ). Thus, these adipokines and cytokines can also induce cardiac dysfunction and promote the formation of atherosclerotic plaques ( 19 ). Obesity is also associated with traditional cardiovascular risk factors such as insulin resistance, DM, hypertension, and dyslipidaemia that increase the CVD risk ( 2 ).

Conflicting results have been reported in studies evaluating arterial stiffness in obese individuals. In the first study in which arterial stiffness was evaluated in obese individuals, it was determined that arterial stiffness increased in obese patients and that increased arterial stiffness was independent of age, sex, and blood pressure levels. However, in that study, both obese and nonobese subjects were selected from individuals with known essential hypertension ( 20 ). Several epidemiological and clinical studies after that initial study revealed that obesity is associated with increased arterial stiffness ( 21 – 25 ). In addition, some studies in this context have revealed an improvement in arterial stiffness as a result of weight loss ( 26 – 27 ). In contrast, some studies to date have suggested a negative relationship between obesity and arterial stiffness ( 28 – 29 ), whereas other studies have suggested that no relationship exists between obesity and arterial stiffness ( 30 – 34 ). In some of these studies, obese subjects with traditional cardiovascular risk factors such as hypertension ( 20 , 33 ) and both hypertension and DM ( 23 , 29 ) were evaluated. In addition, some of those studies evaluated obese subjects with CVD ( 30 – 31 ), whereas others evaluated obese patients with a BMI > 30 ( 24 , 32 , 34 ) or who had not been evaluated for BMI. In our study, obese patients without known traditional CVD risk factors were evaluated, and the group with BMI ≥ 40 and the group with BMI ≥ 30 but < 40 were evaluated separately. According to the results of our study, when we evaluated all obese patients together, we found that arterial stiffness increased in obese patients and that increased arterial stiffness was associated with age, BMI, SBP, DBP, HbA1c, and serum LDL-C levels. When we evaluated the group with BMI ≥ 40 and the group with BMI ≥ 30 but < 40 separately, we found that arterial stiffness was increased in the group with BMI ≥ 40, and increased arterial stiffness was associated with age, SBP, and HbA1c levels. However, we found that there was no increase in arterial stiffness in the group with BMI ≥ 30 but < 40.

In most previous studies evaluating CIMT levels in obese patients, CIMT levels were found to be higher in obese patients ( 35 – 37 ), whereas they were found to be lower in some studies ( 38 ). In addition, one of the studies suggested that weight loss is associated with a significant reduction in CIMT ( 39 ). When we evaluated all obese patients together in our study, we found that CIMT levels in obese patients were similar to those of the control group. When we evaluated the group with BMI ≥ 40 and the group with BMI ≥ 30 but < 40 separately, we found that CIMT levels in the group with BMI ≥ 40 were higher than those of the control group, and CIMT levels in the group with BMI ≥ 30 but < 40 were similar to those of healthy controls.

To predict CVD risk status, biomarkers have been investigated in addition to measurements such as PWV and CIMT. Endocan and ADAMTS are also among the biomarkers that have been investigated for this purpose. In some studies, it has been shown that high endocan levels may be closely related to the development and progression of CVD ( 5 – 6 ). In contrast to these studies, other studies have found that endocan levels are lower in overweight and obese women than they are in lean women, as well as in individuals with metabolic syndrome compared to healthy individuals ( 9 – 10 ). When we evaluated all obese cases together in the current study, we found that endocan levels were lower in obese patients than they were in the control group. When we evaluated the group with BMI ≥ 40 and the group with BMI ≥ 30 < 40 separately, we found that the endocan levels in the group with BMI ≥ 40 were similar to those of the control group, in the group with BMI ≥ 30 < 40, endocan levels were lower than those of the control group. In our study, we could not find a relationship between endocan levels and CVD risk indicators such as PWV and CIMT measurements. We found that endocan levels were low in all obese patients and the group with BMI ≥ 30 < 40, similar to previous studies with overweight-obese patients and individuals with metabolic syndrome. The results of both our study and previous studies suggest that obesity may be associated with decreased endocan formation in adipose tissue and decreased endocan levels in plasma.

ADAMTSs are involved in the degradation of extracellular matrix proteoglycans such as versican, brevican, and agrecan. It is known that the degradation of versican by ADAMTSs is important in the pathogenesis of atherosclerosis. Studies have shown that serum levels of ADAMTS7 and ADAMTS9 are associated with CVDs ( 11 – 12 ). One of the most important factors that causes obesity is leptin deficiency or leptin resistance. However, most obese individuals have high circulating leptin levels due to leptin resistance. It has been determined in some studies that leptin increases the ADAMTS-4, −5, and −9 gene expression ( 40 , 41 ). When we evaluated all obese patients together in our study, we found that ADAMTS7 and ADAMTS9 levels in obese patients were similar to the control group. When we evaluated the group with BMI ≥ 40 and the group with BMI ≥ 30 < 40 separately, we found that ADAMTS7 and ADAMTS9 levels were similar to the control group in both the group with BMI ≥ 40 and in the group with BMI ≥ 30 < 40. Leptin levels were not measured in our study, but it is known that leptin levels increase in most of the obese cases. Accordingly, it is thought that ADAMTS7 and ADAMTS9 levels will increase in obese patients. However, in our study, an increase in ADAMTS7 and ADAMTS9 levels was not detected in obese patients.

The limitations of our study are the small sample size, noninvasive measurement of PWV, inability to measure leptin levels, and measurement of serum endocan, ADAMTS7, and ADAMTS9 levels by ELISA method.

In conclusion, in our study, we found that arterial stiffness and CIMT increased in the group with BMI ≥ 40, and increased arterial stiffness was associated with age, SBP and HBA1C. We found that there was no increase in arterial stiffness or CIMT in the group with BMI ≥ 30 but < 40. In addition, we found that endocan levels were lower in obese patients than in nonobese individuals, and ADAMTS7 and ADAMTS9 levels were not different in obese patients from nonobese individuals. In this context, more comprehensive studies with larger sample sizes are needed.
